# Retraction notice for: “Ginsenoside Rg1 protects human renal tubular epithelial cells from lipopolysaccharide-induced apoptosis and inflammation damage” [Braz J Med Biol Res (2018) 51(2): e6611]

**DOI:** 10.1590/1414-431X20206611retraction

**Published:** 2020-08-17

X.J. Ni, Z.Q. Xu, H. Jin, S.L. Zheng, Y. Cai, and J.J. Wang

Transplantation Center, The First Affiliated Hospital of Wenzhou Medical University, Wenzhou, China

Correspondence: J.J. Wang: <wangjinjun5566@126.com>

Retraction: Braz J Med Biol Res | doi: http://10.1590/1414-431X20176611 | PMID: 29267498 | PMCID: PMC5731327

The Editors of the Brazilian Journal of Medical and Biological Research (BJMBR) became aware of a denouncement published by independent journalists from the “For Better Science” website including this paper. This denouncement consisted of potential data falsification and/or inaccuracy of results in western blots and flow cytometry plots.

After contacting the Authors and per consensus between the Editors-in-Chief of the BJMBR and the Authors, this article has been retracted.

